# Cell-free DNA: a metabolic byproduct with diagnostic and prognostic potential in rheumatic disorders

**DOI:** 10.3389/fphar.2025.1537934

**Published:** 2025-02-11

**Authors:** Fancheng Liu, Yazhen Su, Xinling Liu, Li Zhao, Zewen Wu, Yang Liu, Liyun Zhang

**Affiliations:** ^1^ Third Hospital of Shanxi Medical University, Shanxi Bethune Hospital, Shanxi Academy of Medical Sciences, Tongji Shanxi Hospital, Taiyuan, China; ^2^ Shanxi Province Clinical Research Center for Dermatologic and Immunologic Diseases (Rheumatic Diseases), Shanxi Bethune Hospital, Taiyuan, China; ^3^ Third Clinical College, Shanxi University of Chinese Medicine, Jinzhong, China

**Keywords:** cfDNA, rheumatic diseases, biomarkers, arthritis, systemic lupus erythematosus

## Abstract

The release of intracellular DNA into the extracellular area occurs via two pathways: cell death and active secretion by cells. The DNA, which is free in the extracellular space, is commonly known as Cell-Free DNA (cfDNA). In healthy people, the levels of cfDNA in the circulation are notably minimal. Within a healthy organism, cfDNA undergoes swift elimination and filtration upon release, ensuring a persistently low concentration in the bloodstream. Conversely, individuals suffering from diverse illnesses like stroke, trauma, myocardial infarction, and various cancers show markedly higher levels of cfDNA in their blood plasma or serum. Further research has shown that cfDNA is associated with a wide range of human diseases and may have a feedback relationship with inflammation, potentially serving as a non-invasive, accurate, sensitive, and rapid biomarker for clinical applications in disease differential diagnosis, activity monitoring, and prognosis assessment. Studies dating back to the 1970s have indicated elevated cfDNA concentrations in SLE. Currently, increased levels of cfDNA are noted in a range of rheumatic disorders. Inflammatory damage in patients with rheumatic diseases promotes the release of cfDNA, while potential abnormalities in cfDNA metabolism further increase its levels. Elevated concentrations of cfDNA are recognized by DNA receptors, initiating immune-inflammatory reactions which subsequently accelerate the progression of disease. Reducing excess cfDNA may help improve inflammation. Additionally, several trials have demonstrated a correlation between cfDNA concentrations and the activity of rheumatic diseases, indicating the potential of cfDNA, a novel marker for inflammation, in conjunction with C-creative protein (CRP), Erythrocyte Sedimentation Rate (ESR) to monitor disease activity in rheumatic conditions. This paper provides an overview of cfDNA and summarizes current research advancements in cfDNA in rheumatic diseases, aiming to offer new perspectives for researchers.

## 1 Introduction

**FIGURE 1:**
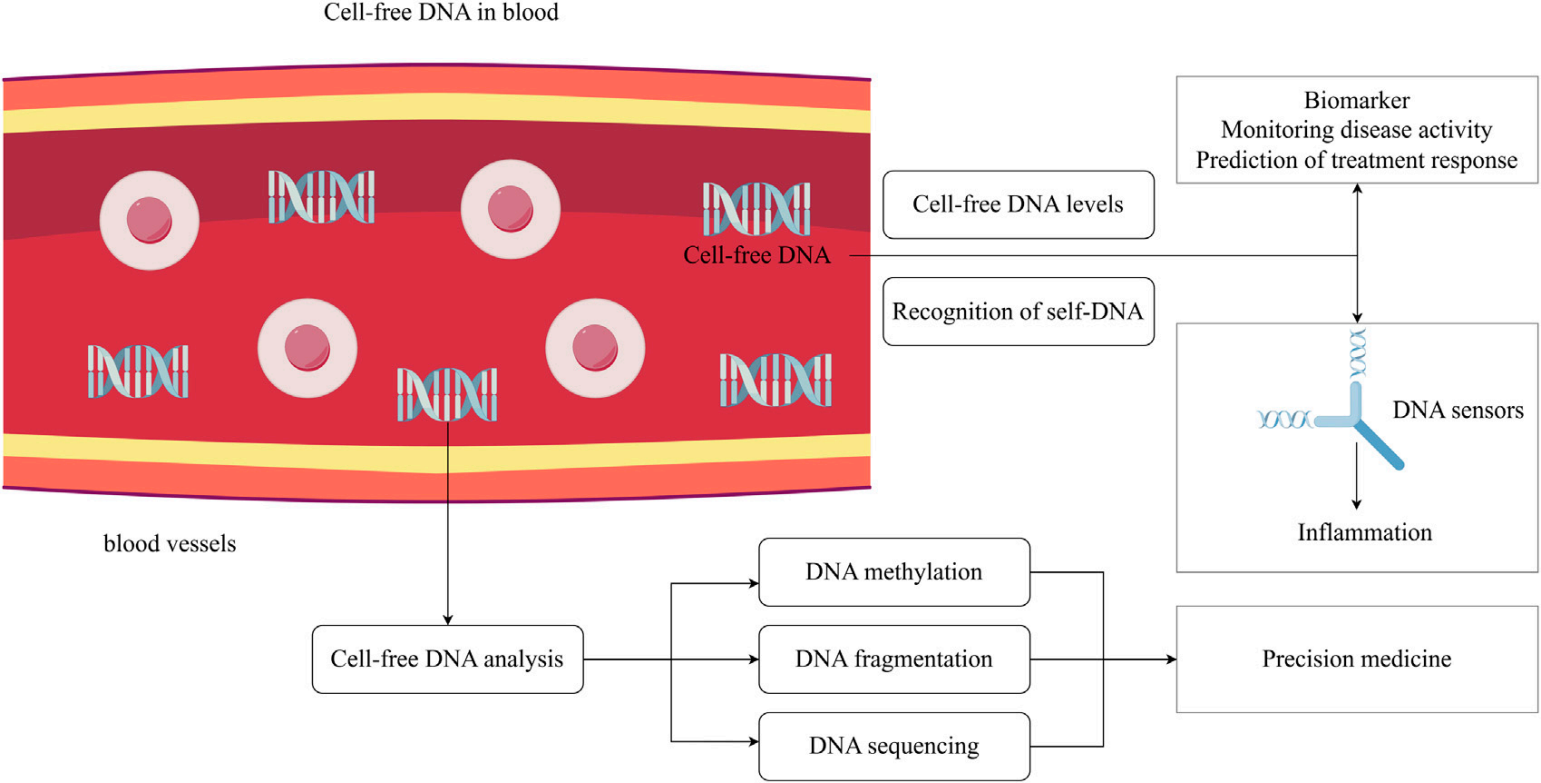
The potential clinical applications of cell-free DNA. Cell-free DNA is present in human circulation, and various studies suggest that the concentration of cell-free DNA may be related to disease activity. Cell-free DNA can be recognized by DNA sensors, triggering immune-inflammatory responses. Metabolite cell-free DNA exhibits different molecular characteristics under different physiological conditions, and its sequences, fragment distribution and methylation can reflect the pathophysiology of body. Analyzing cell-free DNA profile is beneficial for the implementation of precision medicine. The figure is made using Figdraw.

cfDNA is a type of extracellular double-stranded deoxyribonucleic acid fragment that is released during cell death and can be detected in a range of bodily fluids, including serum, plasma, urine, cerebrospinal fluid, saliva, and bronchial lavage. Its concentration is typically very low under normal physiological conditions. Studies have demonstrated that cfDNA levels are elevated in individuals with cancer compared to healthy individuals, as well as in those with inflammatory disorders and tissue damage. Elevated cfDNA levels in patients may be related to heightened apoptosis, necrosis, and impaired clearance of cellular debris associated with the disease, positioning cfDNA a potential indicator of disease activity. Apart from concentration, additional facets of cfDNA such as the ratio of fragment length, methylation pattern, and alteration in gene profile, serve to track the condition of disease. cfDNA liquid biopsy, as a non-invasive diagnostic tool, is widely used in clinical practice today, including prenatal detection of fetal chromosomal abnormalities, cancer screening and phenotyping, and cardiovascular disease diagnosis (Pös et al., 2018; Ranucci, 2019; Dao et al., 2023). In the field of rheumatology, conditions such as Rheumatoid Arthritis (RA), Systemic Lupus Erythematosus (SLE), Systemic Sclerosis (SSc), and primary Sjögren’s syndrome (pSS) exhibit increased cfDNA levels (Vakrakou et al., 2017; Nandi et al., 2020), which correlate with disease activity in patients (Abdelal et al., 2016). This highlights the potential of cfDNA as a diagnostic indicator for a spectrum of rheumatic conditions. Advances in nucleic acid testing technology and the understanding of DNA receptors’ roles in inflammation and rheumatic diseases have further propelled cfDNA research (Figure 1). This review aims to provide a detailed introduction to cfDNA and summarize its ability to reflect disease status changes, thereby deepening understanding of cfDNA in rheumatic diseases.

## 2 Overview of cfDNA

cfDNA fragments range from 70 to 200 base pairs (bp) in length (Cicchillitti et al., 2017). The size of these molecules depends on the mechanisms of their fragmentation. Typically, the peak length is around 166 bp, corresponding to DNA fragments associated with nucleosomes (Snyder et al., 2016). cfDNA can also form complexes by binding with certain proteins and membrane structures.

### 2.1 Release of cfDNA

White blood cells serve as the main source of cfDNA, comprising approximately 76% of plasma cfDNA, with neutrophils being the dominant type (Mattox et al., 2023). cfDNA is highly fragmented, and researchers suggest its origin is primarily related to mechanisms of cell death (Van Der Vaart and Pretorius, 2007). cfDNA release occurs during apoptosis, necrosis, the formation of Neutrophil Extracellular Traps (NETs), and pyroptosis. The apoptosis plays a crucial role in the physiology of eliminating aging, impaired cells and preserving cellular balance (Liu and Pope, 2003). Additionally, it serves as the primary origin of cfDNA in both healthy individuals and patients (Delgado et al., 2013). In the process of apoptosis, nucleosome DNA undergoes programmed enzymatic breakdown, resulting in double-stranded DNA fragments of consistent size (Samejima and Earnshaw, 2005). In contrast, during necrosis, chromatin undergoes non-specific digestion, leading to the release of larger DNA molecules (Aucamp et al., 2018). One of the factors determining whether a cell undergoes apoptosis or necrosis is the intracellular ATP concentration. In patients with SLE, CD4^+^ T cells exhibit sustained mitochondrial hyperpolarization (Chen and Tsokos, 2022), while in RA patients, naive CD4^+^ T cells in the joint show mitochondrial integrity defects (Wu et al., 2021). Both conditions lead to ATP depletion, thereby triggering necrosis and promoting cfDNA release. NETs are nuclear-derived, decondensed DNA mesh-like structures coated with histones, granular proteins, and cytoplasmic proteins. The formation of NETs is accompanied by the death of neutrophils, which is a unique form of programmed cell death in neutrophils, known as NETosis (Vorobjeva and Chernyak, 2020). Pyroptosis represents a different type of cell death, separate from apoptosis and necrosis, marked by lysis reliant on caspases. The procedure induces the activation of cytokines like interleukin and involves an inflammatory response to various infections and stressors, resulting in swift cell death (Purnama et al., 2023). During the process of pyroptosis mediated by cysteine proteases, the destruction of the plasma membrane promotes the release of cfDNA (Fink and Cookson, 2006). Besides undergoing cell death, cells are capable of releasing DNA itself (Hu et al., 2021). cfDNA remains detectable in the cell culture medium’s supernatant even in the absence of cell death caused by stress (Bronkhorst et al., 2016). Presently, two potential mechanisms for active secretion are recognized: the autophagosome-dependent pathway and the extracellular vesicle pathway (Thakur et al., 2014; Jeppesen et al., 2019), including exosomes and microvesicles. It is established that cells secrete cfDNA principally via extracellular vesicles. Furthermore, studies have indicated that a significant portion of cfDNA is likely preserved in the extracellular vesicle biomass (Fernando et al., 2017).

### 2.2 Clearance of cfDNA

The maintenance of low levels of cfDNA in a healthy body relies on a dynamic balance between normal apoptosis and rapid clearance (Kustanovich et al., 2019). The half-life of cfDNA ranges from a few minutes to several hours (Celec et al., 2018; Yao et al., 2016; Diehl et al., 2008). The short half-life facilitates the ‘instantaneous examination’ of cfDNA, allowing for dynamic tracking of disease progression or treatment reactions and immediate evaluation. cfDNA clearance is carried out by specific enzymes, including extracellular deoxyribonuclease I (DNase I), deoxyribonuclease I-like 3 (DNase IL3), and deoxyribonuclease II (DNase II) found in immune cell lysosomes (Han and Dennis Lo, 2021). Among cancer sufferers, there’s an inverse relationship between DNase levels and cfDNA concentration (Alekseeva and Mironova, 2021). Multiple factors influence these enzymes’ capacity to break down cfDNA, including its attachment to certain proteins or the formation of membrane complexes, its state as either free-floating or encased in extracellular vesicles, and the biochemical makeup, structural features, and steadiness of cfDNA originating from diverse sources (Duvvuri and Lood, 2019). Under pathological conditions, excessive cell death can overwhelm the clearance system, disrupting the dynamic balance and leading to the accumulation of cfDNA in the body. Elevated cfDNA levels induce inflammation and the activation of the type I interferon pathway, a phenomenon especially noticeable in individuals with SLE (Arneth, 2019). Therefore, the rapid clearance of cfDNA is crucial for suppressing inflammatory immune responses in the body (Savill and Fadok, 2000).

### 2.3 Pro-inflammatory effects of cfDNA

Numerous studies have demonstrated that cfDNA within the body can be recognized by DNA receptors and has an immunostimulatory effect (Műzes et al., 2022). cfDNA bound to proteins such as antimicrobial peptides and antibodies can enhance its inflammatory potential. In pathological conditions, elevated levels of certain carrier proteins can promote cellular uptake of cfDNA and protect it from degradation. This interaction can amplify inflammatory responses by stabilizing cfDNA, making it more effective at activating immune pathways and perpetuating inflammation. Anti-dsDNA antibodies serve as a key transporter for cfDNA into cells in SLE. By engaging with the Fc γ receptor II (FcγRII), anti-dsDNA antibodies facilitate receptor-mediated DNA endocytosis, allowing cfDNA to enter the endosomal compartment of plasmacytoid Dendritic Cells (pDCs) containing Toll-like Receptor-9 (TLR-9), subsequently initiating a robust activation of the interferon pathway, leading to disease progression in SLE (Bai et al., 2017). The cationic cytokine Interleukin-26 (IL-26), produced by T helper cells (Th)17, attaches to and accumulates cfDNA via its aggregated cationic charges, creating non-soluble particles that resisit breakdown (Meller et al., 2015). And increased concentrations of IL-26-DNA complexes have been observed in individuals suffering from ANCA-associated vasculitis (Anti-Neutrophil Cytoplasmic Antibody-Associated Vasculitis, AAV) and RA.

Oxidation of cfDNA is another important factor in activating its inflammatory potential (Shmarina et al., 2020). Base modifications caused by Reactive Oxygen Species (ROS) lead to structural changes in cfDNA. Compared to non-oxidized DNA, oxidized cfDNA is more stable and resistant to nuclease degradation. The more stable, oxidized cfDNA accumulates in the body and finally leads to the development of pro-inflammatory responses (Gehrke et al., 2013). Among the range of DNA base modifications caused by ROS, 8-oxo-dG stands out as the most abundant and representative (Chiorcea-Paquim, 2022). Studies have shown that the content of 8-oxo-dG fragments in cfDNA is significantly higher than that in nuclear DNA (nDNA) (Filev et al., 2021). In the process of NETosis, there’s a great release of mitochondrial DNA (mtDNA) molecules containing 8-oxo-dG, playing a vital role in the systematic inflammation of SLE (Lood et al., 2016).

The pro-inflammatory potential of cfDNA is also influenced by its intracellular origin. Unlike nDNA, mtDNA is a potent trigger of inflammation (Zhang et al., 2010). mtDNA contains inflammatory, hypomethylated CpG sequences similar to bacterial DNA, which can act as ligands to activate the pattern recognition receptor TLR-9, thereby inducing immune inflammatory responses. Injecting mtDNA or bacterial DNA containing hypomethylated CpG sequences into mouse joints can induce localized inflammation and arthritis, in contrast to the ineffectiveness of nDNA injections (Collins et al., 2004). Furthermore, mtDNA is located near the respiratory chain, and its vulnerability to oxidative damage increases when the mitochondrial respiratory chain generates substantial ROS. Compared to nDNA, mtDNA exhibits higher levels of oxidation (Rong et al., 2021).

**FIGURE 2:**
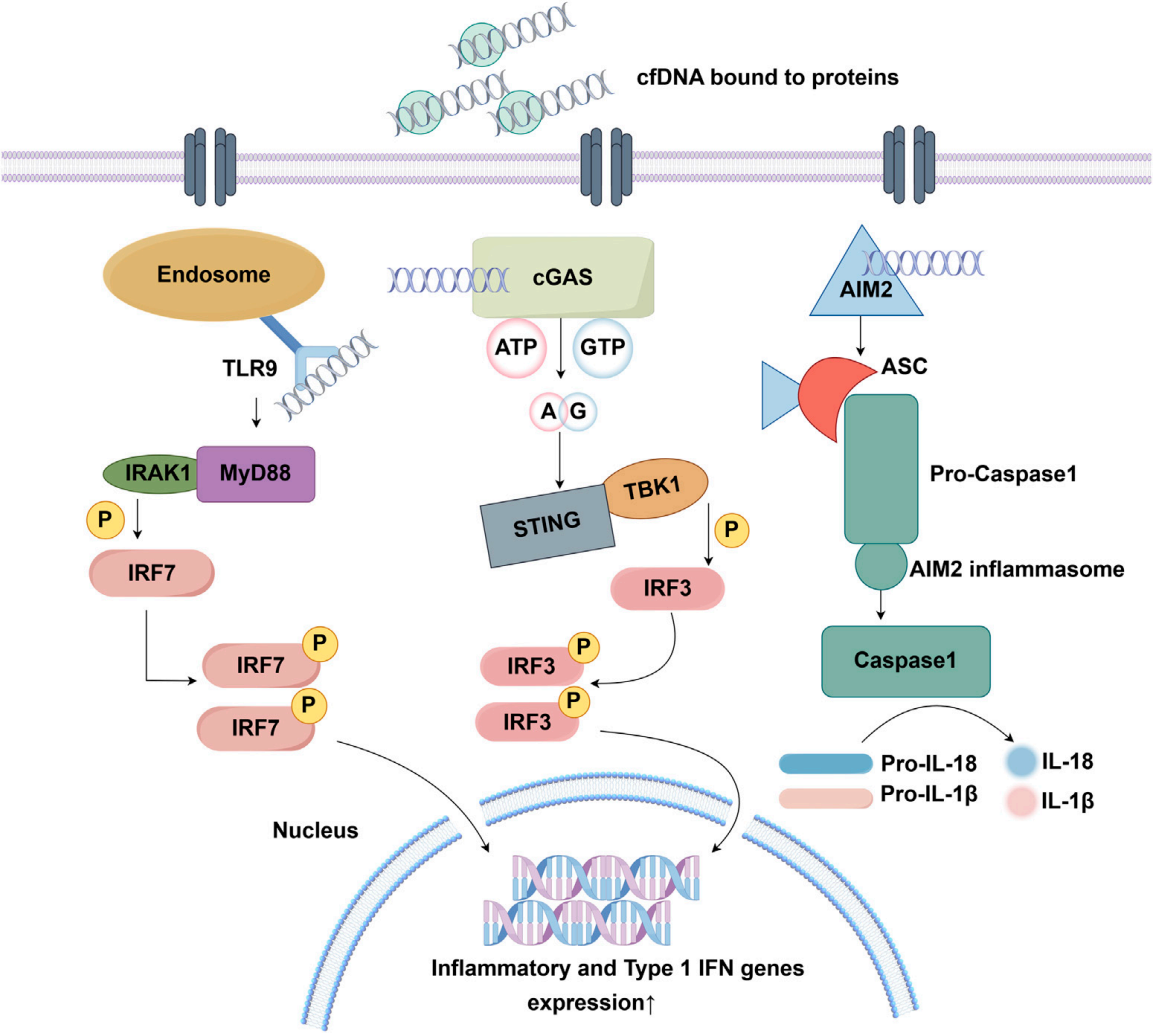
Intracellular immune signaling pathways related to cfDNA. cfDNA has the ability to attach to specific proteins, including antimicrobial peptides and antibodies, creating complexes that enhance cell absorption of cfDNA and safeguard it against breakdown. Upon entering the cell, cfDNA gets identified by DNA receptors like TLR-9, cGAS, and AIM2, triggering signaling pathways and prompting the generation of inflammatory cytokines, which eventually results in inflammation. The figure is made using Figdraw.

cfDNA that escapes from cells and accumulates *in vivo* can be immunostimulatory. DNA sensors like TLR-9, Cyclic GMP-AMP Synthase (cGAS), and Absent In Melanoma 2 (AIM2) can bind to cfDNA and induce immune inflammatory responses (De Luca et al., 2023) (Figure 2). TLR-9 specifically responds to DNA sequences that have unmethylated CpG sites and the activation of the transcription factors Nuclear Factor kappa-B (NF-κB) and Interferon Regulatory Factor 7 (IRF-7) is feasible through the Myeloid Differentiation Primary Response Protein 88 (MyD88)-dependent pathway (Nickerson et al., 2010). The cytoplasmic DNA sensor, cGAS, facilitates the generation of cyclic GMP-AMP(cGAMP) using ATP and GTP (Hopfner and Veit, 2020). Subsequently, cGAMP binds to the Stimulator of Interferon Genes (STING) endoplasmic reticulum adaptor protein, triggering TANK-binding kinase 1 (TBK1) and IκB kinase (IKK). This action results in the phosphorylation of the IRF3 transcription factor, the activation of NF-κB, and the production of various inflammatory cytokines including type I interferons, Tumor Necrosis Factor (TNF), IL-1β, and IL-6. Capable of identifying dsDNA from viruses, bacteria, and mitochondria, the AIM2 inflammasome induces intricate oligomerization and engagement with adaptor proteins like Apoptosis-associated speck-like protein containing a caspase recruitment domain (ASC), prompting the release of IL-1β and IL-18 (Fu et al., 2024).cfDNA acts as a crucial self-antigen and pathogenic factor, and its recognition by DNA sensors promotes the progression of inflammation and is associated with the pathology of various rheumatic diseases.

### 2.4 Immune-related adverse events (irAEs) and cfDNA in cancer

Cancer cells in patients with tumors actively secrete or release large amounts of cfDNA into the bloodstream during apoptosis, and the cfDNA derived from cancer cells is called circulating tumor DNA (ctDNA). ctDNA is highly consistent with the nuclear genes of tumor cells and contains specific mutations corresponding to the tumor. Employing digital Polymerase Chain Reaction (dPCR) and Next-Generation Sequencing (NGS) for evaluating the molecular profile and longitudinal changes of ctDNA can predict the patient’s survival prognosis (Indini et al., 2021). Research indicates that for patients with Non-small-cell lung cancer (NSCLC) undergoing Immune checkpoint inhibitors (ICI) therapy, a reduction in ctDNA levels correlates with better treatment outcomes and survival rates, whereas those with stable ctDNA levels during treatment experience reduced survival periods. Relative to radiographic assessments, alterations in ctDNA manifest sooner and are more reliable. Recent studies conducted by Murray et al. highlights that among metastatic NSCLC patients undergoing immunotherapy, the elimination of cell-free Tumor Load (cfTL), defined as the decrease of the maximum mutant allele frequency to 0% in plasma samples, shows a notable correlation with Progression-Free Survival (PFS) and overall survival (Murray et al., 2024). This is especially helpful in evaluating survival outcomes in patients with radiologically stable disease. Likewise, Jin et al. found comparable outcomes in their research on ctDNA among gastric cancer patients undergoing ICI treatment (Jin et al., 2020). A positive reaction to immunotherapy was linked to the reduction of ctDNA levels observed during the monitoring period. Individuals whose ctDNA levels post-treatment below the detection limit exhibited an extended PFS compared to those whose ctDNA levels were detectable. Furthermore, the ctDNA molecular profile is capable of predicting the resistance of gastric cancer patients to immunotherapy. Specifically, mutations in Transforming Growth Factor-beta Receptor (TGFBR), Ras Homolog Family Member A (RHOA), and Phosphatidylinositol-3,4,5-Trisphosphate Dependent Rac Exchange Factor 2 (PREX2) within ctDNA affect the PFS of immunotherapy. Patients with alterations in the CCAAT Enhancer Binding Protein Beta (CEBPA), Fibroblast Growth Factor Receptor 4 (FGFR4), Mesenchymal-epithelial Transition Factor (MET), or Lysine Methyltransferase 2B (KMT2B) genes face a higher risk of encountering irAEs. As we can see, ctDNA has the utility in identifying genetic alterations and predicting the prognosis, and monitoring progression of cancer. It's evident that cfDNA is useful for detecting genetic changes, predicting the prognosis, and tracking disease development.

## 3 Research progress of cfDNA in RA

RA, an autoimmune disorder, is marked by persistent synovial inflammation and advancing joint harm, potentially resulting in joint abnormalities, functional loss, and a significant incidence of disability and deformities (Weyand and Goronzy, 2021). Timely and effective treatment is crucial for controlling inflammation, preventing disease progression, functional impairment, and adverse outcomes. The detection of Rheumatoid Factor (RF) and Anti-Citrullinated Peptide Antibodies (ACPA) shows good sensitivity and specificity in the early diagnosis of RA, but autoantibodies may share epitopes, and some non-RA rheumatic diseases or chronic infections may also present with positive RF or anti-CCP antibodies (Taylor et al., 2011). Some RA patients may also present with negative RF and anti-CCP antibody results (Jang et al., 2022). Serological diagnosis may have false negatives and false positives. Moreover, the diverse pathogenesis of RA leads to significant patient heterogeneity, and some patients may not respond well to treatment. Therefore, suitable biomarkers need to be developed to optimize serological diagnosis and guide clinical treatment strategies (Zhao and Li, 2018).cfDNA, which has garnered attention in oncology, is an ideal candidate for developing new biomarkers.

Research indicates increased levels of cfDNA in the peripheral blood and synovial fluid in patients with RA. Dong et al. evaluated the levels of cfDNA in the plasma and synovial fluid of 80 patients with RA and a control group, utilizing Picogreen technology. The findings showed a significant difference in plasma cfDNA concentrations between individuals with RA and those without. Unexpectedly, the average concentration of cfDNA in the synovial fluid of patients with RA was about 77 times greater than that in plasma (Dong et al., 2020). Moreover, cfDNA concentration correlates with RA disease activity. Rykova et al. found that RA patients exhibiting greater disease activity showed elevated median levels of plasma circulating nDNA and CRP, in contrast to those with lower disease activity and the control group (Rykova et al., 2017). And, following the application of biological Disease-modifying Anti-rheumatic Drugs (bDMARDs), a clear positive correlation emerged between plasma cfDNA concentration and DAS28 scores in RA (Macáková et al., 2022). However, the correlation between synovial fluid cfDNA and plasma CRP was not strong (Birkelund et al., 2020). Also, Macáková et al. analyzed the relationship between RF, ACPA concentrations, and cfDNA, finding no significant correlation. This implies that cfDNA might depend on different inflammatory mechanism. Combining these biomarkers could potentially enhance the serological diagnosis of RA.

**FIGURE 3:**
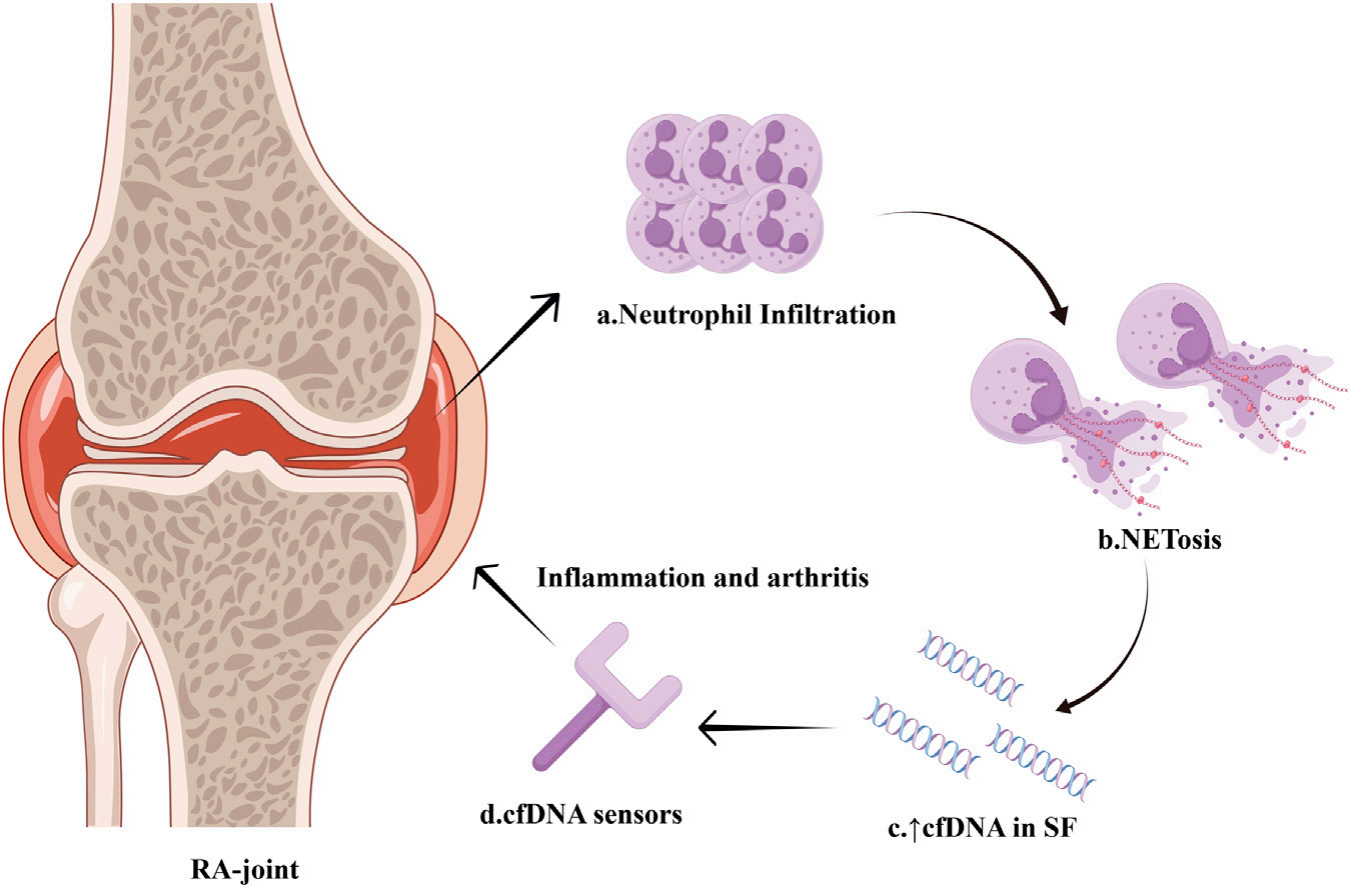
Intense NETosis within the synovium of RA-joint. Infiltration of neutrophils occurs within the synovium of RA patients, and a large number of neutrophils undergo NETosis. The massive formation of NETs leads to a significant increase in cfDNA concentration in the synovial fluid. Sensors for nucleic acid are capable of identifying high levels of cfDNA and triggering inflammation pathway, thereby accelerating synovitis development. The figure is made using Figdraw.

RA synovial fluid contains a notably greater amount of cfDNA compared to plasma, indicating that the release of cfDNA in RA mainly occurs in the joints and is probably linked to inflammation (Dong et al., 2020). Compared to other types of arthritis, RA synovial fluid cfDNA levels are 38.8 times higher than in osteoarthritis patients, while there is no statistically significant difference in intra-articular cfDNA levels between RA and spondyloarthritis patients (Birkelund et al., 2020). Analysis of the protein composition in RA synovial fluid reveals that neutrophil-derived proteins are predominant. This finding is consistent with RA’s pathogenesis, as there is a significant infiltration of neutrophils in the synovial fluid of RA patients. Furthermore, NETs-related proteins from neutrophils are associated with synovial cfDNA levels. These observations suggest that cfDNA in the synovial fluid predominantly originates from NETosis. The intense neutrophil-mediated inflammation and NETosis contribute to the high levels of cfDNA observed in RA synovial fluid (Wright et al., 2021) (Figure 3). In turn, high levels of cfDNA in synovial fluid could serve as a marker for neutrophil death and NETs formation within the joint. The quantification of synovial cfDNA therefore may be utilized as a diagnostic tool for severe arthritis and potentially help in identifying and monitoring the extent of inflammation in RA.

To further clarify the pathological relevance of synovial cfDNA, Dong et al. isolated and collected synovial cfDNA from RA patients and used it to stimulate inflammatory cells *in vitro* (Dong et al., 2020). Flow cytometry analysis revealed an increase in inflammatory cytokine expression in inflammatory cells following stimulation with synovial cfDNA. Additionally, it was discovered that serum from patients with RA triggers Gasdermin D (GSDMD)-dependent pyroptosis in monocytes, linked to increased cfDNA levels in the peripheral blood (Wu et al., 2020). The findings validate the inflammatory properties of cfDNA in RA. And the inflammatory properties of cfDNA in RA are attributed to its low-methylated CpG sequences. Through genomic sequencing of the synovial cfDNA in patients, it was found that, compared to OA patients, cfDNA from RA patients has a higher density of CpG sequences, with the methylation level of CpG sequences significantly lower than that in OA patients. The TLR9 receptor primarily identifies these inflammatory low-methylated CpG sequences as ligands, which exert a strong inflammatory impact. In cells stimulated by cfDNA from RA, NF-κB nuclear translocation induced by triggering of the TLR9 pathway could be observed (Hashimoto et al., 2023). Cell analysis shows that RA CpG-motif-rich (CMR) sequence has a potent ability to activate inflammatory cells to produce the inflammatory mediator TNF-α, while using smallinterferingRNA (siRNA) to suppress TLR-9 markedly reduces the effect of CMRs in inducing TNF-α.

Abnormal release of cfDNA activates innate immune pathways such as TLR-9, leading to chronic inflammation and promoting the advancement of RA. Clearing excess cfDNA could be effective in reducing inflammation and lessening the severity of RA. The innovative RA therapy strategy targeting cfDNA involves administering cationic nanoparticles into the body, using charge interactions to attach to the negatively charged cfDNA, thus removing surplus cfDNA and suppressing immune response (Chen and Tsokos, 2022; Peng et al., 2024; Liu, 2003). As scavengers of DNA molecules, cationic nanoparticles markedly reduced arthritis symptoms in rats; yet, their potent positive charge could lead to systemic toxicity in intravenous blood flow, and their effectiveness is hindered by biocompatibility, serum tolerance, and subpar clearance. For this, researchers altered the cationic nanoparticles chemically to precisely manipulate their molecular composition, with the goal of harmonizing toxicity, enhancing molecular binding capacity, and boosting biodistribution. In the Collagen-Induced Arthritis (CIA) rat model, the modified cationic dendritic polymers successfully reduced symptoms like joint inflammation, increased synovial growth, and bone damage.

On the other hand, cfDNA might serve as a marker to predict the treatment response of bDMARDs in RA. Previously noted, patients with RA exhibit increased levels of cfDNA in their blood. However, Dunaeva’s research revealed a contrasting result, showing that the serum cfDNA concentrations in patients with early-stage RA are comparable to those in healthy individuals, whereas in patients with late-stage RA, serum cfDNA levels are markedly less than those in early-stage RA patients and healthy subjects (Dunaeva et al., 2015). The authors asserted that the fluctuations in cfDNA levels are not caused by variations in DNase activity and hypothesized that the decrease in cfDNA levels in RA patients may be related to the treatment they received. Indeed, the use of medicine can affect cfDNA levels, with report indicating that in 7 out of 10 patients, plasma cfDNA levels changed significantly (either rising or falling) within an hour after infliximab infusion (Zhong et al., 2007). The findings of Hashimoto’s study reveal that RA patients undergo alterations in cfDNA levels post-treatment with bDMARDs, linked to improved disease activity (Hashimoto et al., 2017). Subsequent studies have confirmed that bDMARDs like tocilizumab may possess strong DNase activity, directly degrading cfDNA and thereby inhibiting the inflammatory response induced by the TLR-9 pathway (Hashimoto et al., 2023). To analyze the impact of bDMARDs on cfDNA in RA patients and clarify the correlation between cfDNA level changes and treatment response, Macáková et al. collected blood samples from RA patients before starting bDMARDs treatment, as well as at 3 months and 12 months after treatment initiation. The data showed that, after 3 months of bDMARDs treatment, extracellular DNA in responders decreased significantly by 60%, with extracellular-nDNA decreasing by 58%. In contrast, plasma extracellular DNA levels remained unchanged in the moderate response and non-responding groups (Macáková et al., 2022). Macáková holds the view that plasma extracellular DNA, particularly nuclear-derived extracellular DNA, has the potential to monitor RA treatment response. Conversely, Rykova and Laukova possess different views. Rykova found no significant correlation between the plasma cfDNA of RA patients and the duration of treatment after the adoption of a unified therapy combining methotrexate, etoricoxib, and folic acid. Another group of patients received combined treatment with rituximab and methotrexate, and there was no difference in cfDNA levels between the two treatment groups (Rykova et al., 2017). The findings of Laukova’s study revealed a 3-month postponement in the decrease of plasma cfDNA in RA sufferers after bDMARD treatment, in contrast to the clear improvement in clinical manifestations and laboratory parameters. Consequently, Laukova and colleagues are of the opinion that cfDNA does not serve as a reliable indicator of disease activity (Lauková et al., 2018). Due to variations in cfDNA detection intervals, patient health histories, and therapeutic medications, a direct comparison of the findings is not feasible. Post-treatment cfDNA levels may be influenced by various factors, including the timing of blood draws and the types of bDMARDs used. Therefore, to understand the relationship between cfDNA level changes and responses to different bDMARDs treatments, long-term dynamic monitoring and the inclusion of more trial samples are necessary.

To summarize, RA patients frequently exhibit increased cfDNA levels, which appear to correlate with the disease’s progression and the effectiveness of treatment (see summary in Table 1). Consequently, cfDNA could be an effective parameter for tracking RA’s disease progression and forecasting future treatment outcomes. There’s no significant connection between cfDNA and the levels of serological markers, likely because they depend on separate inflammatory mechanisms. However, cfDNA could bridge the diagnostic divide for patients with seronegative RA, regardless of serological factors. Large-scale longitudinal research remains essential to document the evolving alterations of cfDNA in RA throughout the disease’s development and following treatment. Additionally, while clearing excess cfDNA can significantly alleviate joint inflammation, further optimization and testing are required before cfDNA can be implemented in clinical practice. It is anticipated that cfDNA will soon become a new tool in the clinical management of RA.

**TABLE 1:** Research progress of cfDNA in RA.

Study	Material	Subjects	Results
Dong et al. (2020)	Plasma and SF	50 HC, 80 RA, and 33 OA	↑plasma cfDNA of patients SFcfDNA in RA 38.8-fold higher than OA,SFcfDNA ∼77-fold higher than plasma cfDNA in RA
Rykova et al. (2017)	Plasma	63 HC and 74 RA	↑plasma n-cirDNA in RA than HC Significant correlation between n-cirDNA and disease activity No correlation between cfDNA and therapy duration No difference in cfDNA between two groups received different treatment
Macáková et al. (2022)	Plasma	40 RA	↓ecDNA and nuclear ecDNA in good responders after treatment, no changes of ecDNA in moderate and non-responders Positive correlation between ecDNA and DAS28 after treatment, no correlation with the RF, ACPA
Birkelund et al. (2020)	SF	32 RA and 24 SpA	No difference in SFcfDNA between the RA and SpA Correlation between cfDNA and NETs proteins
Dunaeva et al. (2015)	Serum	29 HC, 39 eRA, 26 esRA and 33 RRMS	↓cfDNA observed in esRA in comparison with HC, eRA and RRMS No difference in cfDNA between HC, eRA and RRMS DNase activity similar in the sera of all tested groups
Hashimoto et al. (2017)	Plasma	21 HC and 30 RA	↑ccfDNA in good responders until 8 weeks, while those of moderate or no response not
Lauková et al. (2018)	Plasma	32 RA	Both DAS28 and CRP decreased after 3 months of treatment, plasma ecDNA decreased significantly only after 6 months

HC: healthy control; SF: synovial fluid; OA: Osteoarthritis; RA: Rheumatoid arthritis; cfDNA: cell-free DNA; n-cirDNA: nuclear circulating DNA; ecDNA: extracellular DNA; DAS28: disease activity score; RF: Rheumatoid factor; ACPA: Anti-citrullinated protein antibody; SpA: Spondyloarthritis; NETs: Neutrophil extracellular traps; eRA: early RA; esRA: established RA; RRMS: relapsing-remitting multiple sclerosis; ccfDNA: circulating cell-free DNA.

## 4 Research progress of cfDNA in SLE

Patients with SLE exhibit elevated cfDNA levels in contrast to healthy individuals, yet the link between cfDNA concentrations and SLE disease progression is still debated. The majority of research indicates a direct correlation between cfDNA amounts and SLE disease activity. To determine the correlation between SLE disease activity and cfDNA levels, Xu et al. compared cfDNA levels between 18 patients with high SLEDAI scores and 40 patients with low SLEDAI scores. They found that the median cfDNA levels were significantly higher in patients with active disease compared to those with inactive disease. Additionally, there was a noticeable pattern in which elevated cfDNA levels correlated with increased SLEDAI scores among all patients, including those who were not pregnant and those who were (Xu et al., 2018). However, Atamaniuk and Tug found no correlation between cfDNA levels and SLEDAI scores in their studies (Tug et al., 2014; Atamaniuk et al., 2011). Despite this, a series of plasma measurements in SLE patients suggest that fluctuations in cfDNA levels can partially reflect changes in disease status, particularly disease progression. Indeed, studies have reported conflicting results regarding the relationship between cfDNA and inflammatory markers like CRP, as well as complement components C3 and C4. Some research indicates a correlation, while others find no significant link (Tug et al., 2014; Xu et al., 2018; Hendy et al., 2016). Xu et al. further investigated whether the inflammatory process was associated with higher cfDNA levels by examining other inflammatory markers. They found that reductions in C3 and C4 levels were not associated with increases in cfDNA levels. In Tug’s study of the relationship between cfDNA levels and individual clinical parameters, it was found that cfDNA levels showed a significant correlation with C3 and C4 in both SLE patients and healthy controls. Additionally, in the healthy control group, cfDNA was significantly correlated with CRP, whereas no such correlation was observed in SLE patients. In Hendy’s correlation study, cfDNA levels were significantly negatively correlated with C3, positively correlated with serum CRP levels, and showed no correlation with age, sex, SLEDAI score, or C4. These conflicting results may be due to differences in methodologies and clinical diversity among patient populations. The levels of C3, C4, and CRP can vary over time, so results may differ based on the timing of assessments. Although the results are not uniform, cfDNA levels in SLE show a strong correlation with medical assessments. Elevated cfDNA levels reflect the inflammatory state, suggesting that cfDNA could potentially be considered an acute-phase reactant in SLE.

Aside from inflammation markers, cfDNA levels are also correlated with autoantibody titers. Atamaniuk’s study found a statistically significant correlation between cfDNA and anti-histone antibodies in SLE patients, while Hendy discovered that cfDNA levels were associated with the disease severity marker, anti-nucleosome antibodies. On the other hand, it is unexpected that Hendy’s correlation analysis found a negative correlation between the levels of cfDNA and ANA, anti-dsDNA levels, whereas Xu did not find a correlation between dsDNA antibodies and cfDNA (Atamaniuk et al., 2011; Xu et al., 2018; Tug et al., 2014). In theory, cfDNA is likely the initiating factor for the production of anti-dsDNA in SLE, and there should be a certain positive correlation between the two. For this anomalous phenomenon, researchers thought that in patients with high autoantibody titers, the formation of DNA-antibody immune complexes may directly interfere with cfDNA detection or accelerate the clearance of DNA-antibody complexes (Truszewska et al., 2017). Continuous sampling might provide a better understanding of the association between cfDNA levels and anti-dsDNA antibody levels.

Apart from differences in levels, the cfDNA molecular characteristics in SLE patients also differ from those in healthy individuals (Chan et al., 2014). Rebecca W. Y. Chan conducted a high-resolution analysis of the biological characteristics of plasma DNA in SLE patients using large-scale parallel genomics and methylomics sequencing, and discovered numerous differences between the plasma DNA of SLE patients and those of healthy individuals. Firstly, the data obtained from sequencing studies indicated that the cfDNA profiles in SLE patients contained multiple measured genomic representations (MGRs) that were either under-expressed or over-expressed, which were not present in healthy controls. Researchers hypothesize that in SLE, anti-DNA antibodies may preferentially bind to specific cfDNA sequences to form complexes, with the cfDNA protected in these antibody-bound regions, leading to an increase in genomic representations in certain areas and a decrease in those not preferentially bound by antibodies, resulting in the emergence of abnormal MGRs. This phenomenon has currently only been observed in SLE, and the significance of the abnormal MGRs in SLE remains unclear. Secondly, the distribution of cfDNA fragment lengths varies between patients with SLE and healthy individuals. In healthy individuals, the plasma cfDNA molecular size is predominantly centered around 166 bp, with a prominent peak at this length in the cfDNA size distribution curve (Shi et al., 2020).In contrast, SLE patients exhibit a reduced peak height at 166 bp, indicating a lower abundance of cfDNA fragments of this size. Moreover, there is an increase in the peak height of smaller cfDNA fragments, particularly those shorter than 115 bp. These changes are more pronounced in patients with active SLE compared to those with inactive SLE. In further research, Rebecca W. Y. Chan defined plasma DNA molecules ≤115 bp as short DNA fragments and measured the percentage of short plasma DNA fragments in each sample. The median percentage of short plasma DNA fragments in the plasma was 10% for healthy individuals, 14% for patients with inactive SLE, and 31% for patients with active SLE. The percentage of short DNA fragments showed a positive correlation with SLEDAI scores and anti-dsDNA antibody levels.

In addition, cfDNA is hypomethylated in SLE. The genome-wide methylation density is significantly lower in patients with active SLE compared to healthy individuals and those with inactive SLE. Active SLE patients have a greater number of hypomethylated regions than those in the inactive group. There is a correlation between the percentage of hypomethylated regions and both SLEDAI scores and anti-dsDNA antibody levels. Research reveals that injecting hypomethylated homologous apoptotic DNA can trigger SLE-like symptoms and cause non-susceptible mice to develop proteinuria and anti-DNA antibodies, indicating that hypomethylated DNA has immunogenic properties and is related to the pathogenesis of SLE (Wen et al., 2007). It is hypothesized that cfDNA hypomethylation may influence disease progression by inducing excessive production of anti-DNA antibodies. Notably, short DNA fragments in the plasma of SLE patients have even lower methylation levels. There is a negative correlation between genome-wide methylation density and the proportion of short DNA fragments (≤115 bp). The decrease in methylation density is more pronounced in the active SLE group compared to healthy individuals and those with inactive SLE, with plasma DNA fragments progressively shortening.

Hypomethylation of DNA plays a crucial role in the molecular process of SLE, with the stable intermediate product of active DNA demethylation, 5-Hydroxymethylome (5hmC), influencing gene expression through gene modification, contributing equally to the development of SLE. In order to explore the 5hmC modification status in cfDNA from SLE patients, Tong et al. conducted bioinformatic analysis of the whole-genome 5hmC profile of plasma cfDNA from healthy controls and SLE patients to identify differential hydroxymethylated regions (DhMR) (Tong et al., 2024). The results showed significant differences in both enrichment and motifs between the plasma cfDNA 5hmC profiles of the SLE and HC groups. Functional enrichment annotation analysis was then performed to further explore the biological processes involved in 5hmC modification in SLE. Compared to the healthy control group, genes related to metabolic and immune pathways in SLE patients exhibited dysregulated 5hmC enrichment, including the Wnt signaling pathway, inflammatory responses, and cytokine-mediated signaling pathways. cfDNA with high DhMRs may be closely associated with SLE. Based on this, the experiment additionally included a group of SLE patients with higher SLEDAI scores to investigate the relationship between 5hmC and disease progression. Compared to the original SLE and HC groups, this subgroup exhibited higher insertion fragment size and multiple enrichment frequencies. The increase in frequency in this subgroup confirms that the level of 5hmC rises with SLE activity, indicating a significant difference in the distribution of 5hmC during disease progression. The above conclusion highlights the potential of 5hmC modifications in cfDNA as a candidate biomarker for the diagnosis and monitoring of SLE.

The differences in cfDNA molecular characteristics between healthy individuals and SLE patients suggest variations in cfDNA sources. Jeremic found that SLE patients have higher levels of NETs-related markers compared to healthy controls. NETs are abnormally increased in SLE patients and become a major source of cfDNA (Jeremic et al., 2019). Low-density granulocytes (LDGs) are a subset of neutrophils with enhanced NETs formation ability. Henning found that, compared to healthy controls, SLE patients have a significantly higher proportion of LDGs and elevated levels of circulating plasma NETs (Henning et al., 2024).In SLE, elevated cfDNA levels may also be due to impaired NETs clearance. Leffler found that patients with active SLE have reduced NETs degradation ability (Leffler et al., 2012). NETs are composed of extracellular chromatin, and their accumulation is primarily regulated by the degradation activity of DNase I and DNase IL3 (Angeletti et al., 2021). A mutation in one of the DNase genes can lead to the formation of anti-DNA antibodies and is associated with severe lupus-like syndromes and lupus nephritis. In SLE, DNase may have inactivating mutations, reducing its activity compared to healthy individuals (Yasutomo et al., 2001). Relevant studies in mouse models also show that mice lacking DNase I display typical symptoms of SLE, including the production of ANA and the accumulation of glomerular immune complexes (Napirei et al., 2000). Additionally, anti-DNase antibodies can impair DNase function. Hartl’s research indicates that reduced DNase IL3 activity in Lupus Nephritis (LN) patients is linked to neutralizing anti-DNase IL3 antibodies, which contribute to increased cfDNA accumulation and anti-DNA autoimmunity in SLE (Hartl et al., 2021). Notably, the lack of DNase correlates with alterations in the jagged ends of cfDNA, and a shortage of DNase IL3 can markedly lower the jagged index in plasma DNA exceeding 166 bp. Studies indicate that in patients with SLE, the DNA’s molecular jagged index exceeding 200 bp is less than that in healthy individuals, and this reduction is more noticeable in those who are active. The potential application of this molecular characteristic could may potentially be used for the differential diagnosis of SLE (Ding et al., 2022).

Inefficient DNA degradation is associated with renal involvement. Previous studies have noted the relationship between plasma cfDNA and LN, but the analysis of cfDNA in LN patients is still in its early stages. Recently, Wang et al. described the characteristics of cfDNA in LN patients. They found that the concentration of plasma cfDNA in LN patients was significantly lower than that in non-LN patients. Moreover, the proportion of short cfDNA fragments was notably higher in LN patients compared to non-LN patients. There was also a significant difference in the average methylation scores between LN patients and non-LN patients (Wang et al., 2022). The lower cfDNA concentration in LN patients may be associated with the presence of anti-dsDNA antibodies. Regarding the correlation between cfDNA and LN disease activity, Zhang et al. found that the average cfDNA concentration was significantly higher in patients with active LN compared to those with inactive LN. Additionally, there was a significant correlation between cfDNA concentration and parameters related to the severity of LN. In the SLE group, cfDNA concentration was positively correlated with 24-h urine protein levels and negatively correlated with albumin levels and endogenous creatinine clearance. These data suggest that elevated cfDNA levels may be associated with LN disease activity (Zhang S. et al., 2014). Compared to the aforementioned studies, Truszewska et al. conducted a more detailed validation of the hypothesis that cfDNA profiles are related to LN clinical features, proposing the use of cfDNA levels, the intracellular mtDNA/cf-mtDNA ratio, the cfDNA fragmentation index, and the presence of 54-149 bp and 209-297 bp fragments as clinically relevant parameters for characterizing cfDNA profiles (Truszewska et al., 2020). Research revealed that SLE patients had an average intracellular mt/cf-mtDNA ratio triple that of healthy individuals, and those with a higher mt/cf-mtDNA ratio had a relative cf-mtDNA copy number linked to proteinuria surpassing 0.5 g/day and LN. The fragmentation index reflects cfDNA integrity, and the results showed that SLE patients had significantly higher fragmentation indices compared to healthy individuals, with a higher fragmentation index associated with a lower estimated Glomerular Filtration Rate (eGFR) in SLE patients. Patients with Class V LN had higher fragmentation indices than those with other types of LN. A variance was observed in the distribution of cfDNA sizes between patients with SLE and healthy controls, where about 30% exhibited fragments ranging from 54 to 149 bp and 209-297 bp, absent in healthy subjects. In addition, patients possessing fragments of 54-149 bp and 209-297 bp exhibit notably elevated fragmentation indices and increased cfDNA concentrations. Further analysis revealed that patients with 209-297 bp fragments have lower eGFR, while the concentration of the 54-149 bp fragment is positively correlated with anti-dsDNA antibody titers, CRP, and SLEDAI. The results suggest that the disruption of the relationship between intracellular and cf-mtDNA, the cfDNA fragmentation index, and the presence of the 54-149 bp and 209-297 bp fragments may be related to disease activity and impaired kidney function, and thus should be considered as parameters for characterizing the cfDNA profile. The maximum observed values of the aforementioned parameters in healthy subjects serve as the threshold for choosing patients whose cfDNA profile mirrors that of healthy persons. Patients with a healthy-like cfDNA profile exhibit significantly higher eGFRs than other patients and show no signs of active arthritis. Such patients do not have indications for kidney biopsy or are diagnosed with class II, III, or II + III LN, whereas those without this profile are diagnosed with class IV, V, or VI LN (including those with types combined with class IV or V) or are kidney transplant recipients. SLE patients whose cfDNA levels, mt/cf-mtDNA ratio, or fragmentation rate exceed twice the upper limit of the healthy cfDNA group and contain fragments of 54-149 bp or 209-297 bp are classified into a distinct cfDNA profile group. Patients with different cfDNA profiles have significantly lower eGFRs, with nearly 74% of these patients diagnosed with class IV, V, or VI LN or being kidney transplant recipients, and none being diagnosed with class II, III, or II + III LN. Therefore, the results indicate that the type of lupus nephritis is associated with the cfDNA profile, and lupus nephritis with a poorer prognosis is clearly linked to a distinct cfDNA profile.

cfDNA is involved in the pathogenesis of SLE and is associated with renal involvement (see summary in Table 2). In SLE patients, cfDNA levels are elevated and fluctuate with disease status and some degree of treatment intervention. However, the association between cfDNA and disease activity or inflammatory markers has only been partially confirmed. The formation of immune complexes may affect data measurement, leading to result deviations. Therefore, whether cfDNA concentration can be used to assess SLE disease activity remains under question. On the other hand, studies have shown specific molecular features of cfDNA in SLE, and its apparent genetic distribution may serve as a new clinical marker for SLE. The mechanisms of cfDNA in SLE warrant further exploration.

**TABLE 2:** Research progress of cfDNA in SLE.

Study	Material	Subjects	Results
Xu et al. (2018)	Plasma	22 non-pregnant and 36 pregnant women with SLE and 60 non-pregnant and 199 pregnant women without SLE	↑cfDNA in SLE, a trend of increased cfDNA levels with higher SLEDAI
Tug et al. (2014)	Plasma	59 SLE and 59 HC, follow-up 27 of the 59 SLE	Significant correlation between cfDNA and C3 and C4 in SLE, inverse correlation between anti-dsDNA-antibodies and cfDNA and between ANAs and longer fragments of cfDNA
Atamaniuk et al. (2011)	Plasma and serum	13 SLE and 13 HC	Significant correlations between cfDNA and anti-histone antibodies
Hendy et al. (2016)	Plasma and serum	52 SLE and 25 HC	Negative correlation between cfDNA and ANA, anti-dsDNA, and C3 respectively, positive correlation between cfDNA and CRP, anti-nucleosome Ab respectively
Chan et al. (2014)	Plasma	24 SLE and 11 HC	Aberrant genomic representation, size shortening and hypomethylation of plasma DNA in SLE Correlation with SLEDAI and anti-dsDNA antibodies
Tong et al. (2024)	Plasma	35 SLE and 32 HC	Distinct differences in 5hmC modification patterns between patients with SLE and HCs, varying with disease progression
Jeremic et al. (2019)	Serum	111 SLE, 35 drug-naïve SLE, 50 HC	↑various NETs-associated markers (DNase I, MPO activity, anti-MPO antibodies and cfDNA) in the sera of SLE
Henning et al. (2024)	Plasma and serum	35 iSLE, 41 SLE and 16 HC	↑proportions of LDGs and circulating plasma NETs in iSLE and SLE
Leffler et al. (2012)	Serum	94 SLE	Patients that failed to degrade NETs had a more active disease and lower levels of C4 and C3 in blood
Ding et al. (2022)	Plasma	sporadic SLE comprising 11 inactive, 13 active, and 10 HC	cfDNA jaggedness differed among the healthy controls and patients with active and inactive SLE
Wang et al. (2022)	Plasma	127 SLE (64 with LN, 63 without LN)	↓cfDNA in LN, may be associated with anti-dsDNA antibodies ↑proportion of short fragments of cfDNA in LN Methylation scores in LN differed significantly from those without LN
Zhang et al. (2014a)	Plasma	54 SLE and 43 HC	↑cfDNA in active LN than inactive LN Positively correlation with the quantitative 24-h urinary protein, LDG and neutrophil and inversely correlation with the albumin level and Ccr
Truszewska et al. (2020)	Plasma	43 SLE and 50 HC	Patients with healthy-like cfDNA profile had more often no indications for kidney biopsy or less advanced LN

SLE: Systemic lupus erythematosus; dsDNA: double-stranded DNA; cfDNA: cell-free DNA; SLEDAI: Systematic lupus erythematosus disease activity index; HC: healthy control; C3: Complement 3; C4: Complement 4; ANA: Antinuclear antibody; CRP: C-reactive protein; Ab: Antibody; NETs: Neutrophil Extracellular Traps; DNase I: Deoxyribonuclease I; MPO: Myeloperoxidase; iSLE: incomplete SLE; LDG: Low-density granulocytes; DNASE1L3: Deoxyribonuclease 1-like 3; LN: lupus nephritis; Ccr: endogenous creatinine clearance rate.

## 5 Research progress on cfDNA in other rheumatic diseases

Ankylosing spondylitis (AS) is characterized by progressive chronic inflammation of the axial joints and surrounding tissues. In late stages, it can lead to ankylosis of the spinal joints, reduced spinal mobility, and severe disability (Ebrahimiadib et al., 2021). Under genetic conditions associated with AS, persistent micro-injuries and chronic inflammation that promote disease progression can lead to increased release of cfDNA into the circulation (Mauro et al., 2021). Therefore, cfDNA may serve as a potential biomarker for assessing disease activity in AS. Peng et al. conducted an in-depth exploration of circulating cfDNA in AS patients and its relationship with clinical disease activity and treatment response (Peng et al., 2024). The study found that cfDNA levels in AS patients were significantly higher than in healthy controls and positively correlated with CRP, Ankylosing Spondylitis Disease Activity Score (ASDAS)-CRP, and neutrophil counts. The use of Nonsteroidal Antiinflammatory Drugs (NSAIDs) in combination with DMARDs or tumor necrosis factor inhibitors can reduce cfDNA levels, and a decrease in cfDNA levels after treatment is associated with a good therapeutic response. Notably, treatment-naive patients with higher cfDNA levels respond better to combined therapy than to NSAIDs alone, while those with lower cfDNA levels show similar response rates to either combined or single NSAIDs treatment. In summary, circulating cfDNA levels are significantly correlated with disease activity and treatment outcomes in AS patients. Additionally, cfDNA levels in treatment-naive patients can predict prognosis for different treatments, making cfDNA an effective biomarker for AS inflammation.

NETs are considered to be closely associated with the pathogenesis of AAV. In this process, ANCA plays a key role in triggering NETs formation, which leads to the release of mtDNA and nDNA, thereby inducing inflammatory response. In last year, Karlsson has conducted an in-depth exploration of the differences in circulating cfDNA levels in AAV patients during active and remission phases (Karlsson et al., 2023). Compared to the remission phase, there is no significant difference in mtDNA or nDNA levels in patients with active disease; nor is there a difference in the ratio of mtDNA to nDNA. Moreover, cfDNA shows no correlation with age, gender, clinical phenotype, ANCA positivity, renal involvement, or Birmingham Vasculitis Activity Score (BVAS). Considering the limited number of participants in this research (16 AAV patients), the lack of correlation between cfDNA and disease activity could be influenced by bias. Conversely, Alam’s research encompassed specimens from 103 individuals suffering from ANCA-related vasculitis (33 in active disease, 70 in remission) alongside 41 non-affected subjects. Research revealed that active AAV patients had elevated plasma mtDNA levels compared to healthy individuals and those in remission, while the nDNA levels in active patients were comparable to those in remission. And a notable increase in the mtDNA/nDNA ratio could be observed among active patients in contrast to those in remission (Alam et al., 2019). Within AAV, there’s an increase in cfDNA levels, and its makeup varies depending on the activity of the disease. During active illness, mtDNA, as opposed to nDNA, shows an increase, possibly due to mtDNA being released during platelet activation and the variance in its clearance compared to nDNA. A subsequent study by Giaglis indicated that mtDNA was associated with BVAS and, in multivariate analysis, correlated with AAV activity. In 27 AAV follow-up patients, alterations in mtDNA correlated with shifts in BVAS, in contrast to CRP or ANCA titers, which demonstrated no link to BVAS variations (Giaglis et al., 2023). Both studies confirm the superiority of mtDNA over nDNA in monitoring AAV activity. However, the application of the mtDNA/nDNA ratio measurement in clinical practice still needs to be clarified.

Similar to SLE, there is an abnormality in NETs metabolism in Idiopathic Inflammatory Myopathies (IIMs) (W. Ma et al., 2023). To verify the pathogenic role of NETs in Dermatomyositis (DM) and Polymyositis (PM), Zhang et al. tested the ability to induce and degrade NETs in plasma samples from 97 DM/PM patients (72 with DM, 25 with PM) and 54 healthy controls. The study found a notable increase in plasma cfDNA levels in IIMs patients compared to healthy individuals, with ILD patients showing higher cfDNA concentrations than those without ILD. Consistent with the cfDNA results,DM/PM patients exhibit a markedly improved capacity to trigger NETs compared to the control group, alongside a significant decrease in degradation capacity and DNase I activity. Notably, patients with concurrent Interstitial Lung Disease (ILD) had even lower DNase I activity and the poorest NETs degradation capacity. In IIMs patients, particularly those with ILD, the reduced DNase I activity means that excessive NETs are not fully degraded, suggesting that abnormal NETs regulation may be involved in the pathogenesis of IIMs and could be a contributing factor to the onset and exacerbation of ILD (Zhang S. et al., 2014). According to reports, plasma DNA can induce the production of pro-inflammatory cytokines by activating the TLR-9 and cGAS/STING signaling pathways, thereby causing lung injury (Zhang et al., 2016; Ryu et al., 2020; Ma et al., 2020). cfDNA, as a significant component of NETs and pro-inflammatory DAMPs, may play a role in the development of ILD at IIMs. These findings suggest that cfDNA is involved in the pathogenesis of IIMs, but the specific relationship between cfDNA and disease activity in IIMs still needs further exploration.

## 6 Conclusion

cfDNA, being a non-intrusive liquid biopsy technique, is somewhat indicative of the activity and treatment efficacy in rheumatic conditions. Nonetheless, numerous problems remain that require attention. Initially, the precise mechanisms behind the release of cfDNA are yet to be clarified. Pinpointing the origins of cfDNA in different rheumatic conditions is crucial for comprehending its application in diagnosing, predicting, or tracking these illnesses. Secondly, cfDNA levels are affected by multiple factors, and a single quantitative test might not be precise for disease diagnosis. In different physiological and pathological states, not only does the level of cfDNA change, but molecular characteristics such as methylation density, gene lineage, fragment size distribution, and others also vary. For example, ctDNA contains tumor mutation genes, so it helps assess tumor progression, prognosis, and aids in targeted therapy. To apply cfDNA diagnostics in the clinical treatment of rheumatic and immune diseases, we need to focus on related qualitative research findings of cfDNA. The genetic profiling of cfDNA in rheumatic diseases will contribute to the realization of personalized precision medicine. Additionally, there is an obvious inconsistency in the types of samples, their processing techniques, cfDNA extraction, cfDNA quantification, and the way cfDNA quantification outcomes are presented and interpreted. And with the emergence of qualitative studies on cfDNA, its complexity has also increased. Crucially, present studies predominantly concentrate on RA and SLE, with limited research on other rheumatic conditions and a scarcity of data. The absence of extended-duration studies and constraints in sample sizes compromise the dependability of the findings. A comprehensive grasp of cfDNA’s role as a biomarker in autoimmune disorders necessitates a methodical scientific structure and joint endeavors in executing extensive, multi-institutional forward-looking trials.
